# Expression of genes encoding non-specific immunity, anti-oxidative status and aquaporins in β-glucan-fed golden mahseer (*Tor putitora*) juveniles under ammonia stress

**DOI:** 10.1016/j.fsirep.2023.100100

**Published:** 2023-06-03

**Authors:** Alexander Ciji, Priyanka H. Tripathi, Anupam Pandey, Md Shahbaz Akhtar

**Affiliations:** ICAR-Directorate of Coldwater Fisheries Research, Anusandhan Bhawan, Bhimtal, Nainital, Uttarakhand 263136, India

**Keywords:** Β-glucan, Ammonia, Immune genes, Anti-oxidative genes, Nitric oxide synthase, Golden mahseer

## Abstract

The study investigated the effects of dietary administration of β-glucan on aquaporins and antioxidative & immune gene expression in endangered golden mahseer, *Tor putitora* juveniles, exposed to ammonia stress. For that, fish were fed experimental diets having 0 (control/basal), 0.25, 0.5, and 0.75% β-d-glucan for five weeks and then exposed to ammonia (10 mgL^−1^ total ammonia nitrogen) for 96 h. Administration of β-glucan differentially influenced the mRNA expression of aquaporins, anti-oxidative, and immune genes in ammonia-exposed fish. For instance, the transcript abundance of catalase and glutathione-s-transferase in gill varied significantly among the treatment groups, with the lowest levels in 0.75% β-glucan fed groups. At the same time, their hepatic mRNA expression was similar. Congruently, transcript abundance of inducible nitric oxide synthase considerably decreased in the β-glucan fed ammonia-challenged fish. Conversely, the relative mRNA expression of various immune genes *viz.,* major histocompatibility complex, immunoglobulin light chain, interleukin 1-beta, toll-like receptors (*tlr4* and *tlr5*) and complement component 3 remained largely unchanged in ammonia-exposed mahseer juveniles that were fed with graded levels of β-glucan. On the other hand, a significantly lower transcript level of aquaporins 1a and 3a was noticed in the gill of glucan-fed fish compared to ammonia-exposed fish that received the basal diet. However, branchial aquaporin 3b remained unaltered. Altogether, this study showed that dietary intake of 0.75% β-glucan improved resistance to ammonia stress to a certain degree, probably through activating anti-oxidative system and reducing brachial ammonia uptake.

## Highlights

• We evaluated β-glucan for alleviating ammonia stress in *Tor putitora.*
• A decrease in transcript level of *inos* was observed in β-glucan-fed ammonia-challenged fish.
• β-Glucan had no effect on most of the immune genes of ammonia-exposed fish.
• Aquaporins 1a and 3a expression decreased in β-glucan-fed ammonia-exposed fish.
• Feeding 0.75% β-glucan improved resistance to ammonia stress in *T. putitora.*

## 1 Introduction

Fish in aquaculture are invariably subjected to a variety of environmental and procedural stressors. Among them, ammonia is the most common environmental stressor especially, in intensive culture systems with high stocking density, since it is the end product of protein catabolism and the primary excretory product of fish [1]. Furthermore, the degradation of leftover feed and nitrogenous fertilizers, along with insufficient water exchanges/filtration, causes ammonia to build up in water [2]. Generally, despite lower feeding rates, accumulation of dissolved inorganic nitrogen is higher at lower temperatures/colder months, when the density of phytoplankton, the major consumer of ammonia, is minimal [3]. Moreover, the efficiency of nitrification is also lesser at lower temperatures [4]. Golden mahseer, being a coldwater fish, is more prone to ammonia toxicity.

Ammonia stress can cause several physiological changes, including growth retardation, osmoregulatory disturbance, organ damage, depression of immune and anti-oxidative status, and eventually mortality in aquaculture species [5,6]. More specifically, at high environmental levels, excretion of ammonia is hindered and/or intake of ammonia from the environment happens, causing ammonia poisoning [1,7]. Earlier studies have indicated that elevated levels of ammonia may produce reactive oxygen species (ROS), reactive nitrogen species (RNS), and nitric oxide (NO^.^), imparting oxidative stress [1,7]. In catfish under hyper-ammonia stress, inducible nitric oxide synthase (inos) induction has been documented, which results in enhanced NO generation [8,9]. To counteract oxidative stress-induced damages, cells possess antioxidant defense systems, comprising catalase, superoxide dismutase, glutathione-s-transferase, etc., that act at varying rates to protect or repair such damages. Hence, changes in the antioxidative enzymes can be used as a possible biomarker of stress [10]. Immunoglobulins, cytokines and toll-like receptors are important molecules in immune function regulation [11]. It is well established that aquaporins play an essential role in water and solute/ammonia movements through membranes facilitating osmotic balance [12,13]. However, the expression patterns of aquaporins under ammonia exposure in fish remain largely unknown.

Considering these physiological impacts, there is necessary to decrease the deleterious effects of ammonia toxicity in fish. Dietary manipulation has attracted interest as a tool to mitigate stress-induced damages [14,15]. For instance, oral administration of feed supplements such as cineole [6], taurine [5,16], inositol [17], seaweeds and herbal extracts [1,18,19] were demonstrated to alleviate the adverse effects of ammonia stress in aquaculture species. β-Glucan is one of the promising immunostimulants used in aquaculture considering its immuno-stimulatory, anti-inflammatory, and anti-oxidant properties [20,21]. Recently, prolonged dietary intake of β-glucan has been proposed to mitigate the detrimental effects of stress and shown to improve the welfare of fish exposed to different stressors [22,23]. However, studies investigating the effect of dietary β-glucan supplementation on the anti-ammonia stress response are limited, particularly in the expression of aquaporins and *inos* genes. Therefore, in the present study, the protective role of dietary β-glucan intake against sub-lethal ammonia stress was investigated in an endangered fish golden mahseer, *Tor putitora*.

## 2 Material and method

### 2.1 Experimental/test diets

Four experimental diets were prepared to include 0 (basal/control), 0.25, 0.5, and 0.75% β-d-glucan. To the basal diet, 0.25, 0.5, and 0.75% of β-d-glucan was supplemented by replacing an equal amount of wheat flour (Table 1). The dietary ingredients were accurately weighed, carefully blended, steam cooked, pelletized (1.5 mm diameter), and dried. The β-d-glucan (β-1,3/1,6-glucan) purchased from M/s Kuber Impex Ltd, Madhya Pradesh, India, was extracted from *Saccharomyces cerevisiae* and has been found to have 70.60% purity. The experiment involved four treatment groups, *viz.* control (0% β-glucan), 0.25% β-glucan, 0.5% β-glucan, and 0.75% β-glucan.

**Table 1:** Ingredients composition of experimental diets (% dry weight basis) fed to golden mahseer juveniles during the experimental trial.

Ingredients	% inclusion			
*Dietary treatment groups*	*Control*	*0.25% β-glucan*	*0.5% β-glucan*	*0.75% β-glucan*
Soybean meal$	10.00	10.00	10.00	10.00
Fish meal^a^	55.00	55.00	55.00	55.00
Wheat flour$	18.14	17.89	17.64	17.39
Fish protein hydrolysate^a^	5.20	5.20	5.20	5.20
Soy lecithin^b^	0.50	0.50	0.50	0.50
Egg albumin powder^b^	1.00	1.00	1.00	1.00
Fish oil^a^	4.00	4.00	4.00	4.00
Vegetable oil#	4.00	4.00	4.00	4.00
Vitamin pre-mix^c^	1.00	1.00	1.00	1.00
Mineral pre-mix^c^	1.00	1.00	1.00	1.00
L-ascorbyl phosphate^b^	0.03	0.03	0.03	0.03
α-tocopheryl acetate^b^	0.03	0.03	0.03	0.03
Betaine hydrochloride^b^	0.10	0.10	0.10	0.10
β-glucan powder (70.60% purity)*	0.00	0.25	0.50	0.75
*Proximate analyses [% dry matter basis]*				
*Moisture*	*4.86*	*5.04*	*5.20*	*4.63*
*Dry matter*	*95.14*	*94.96*	*94.80*	*95.37*
*Crude protein*	*44.37*	*44.03*	*43.54*	*44.12*
*Crude lipid*	*13.93*	*14.57*	*14.31*	*14.05*
*Ash*	*13.40*	*13.14*	*13.38*	*12.86*

a Janatha Fishmeal and Oil Products, Udupi, Karnataka, India.

b Himedia Laboratories, Mumbai, India.

$ Procured from local market.

c Prepared manually and all components from Himedia Ltd Mumbai, India.

# Fortune Edible Oils and Foods, Adani Wilmar Limited, Ahmedabad, India.

⁎ β-glucan powder, sourced from *Saccharomyces cerevisiae,* was procured from M/s Kuber Impex Ltd, Indore, India. The active β−1,3/1,6 glucan content in the powder was 70%. Mineral mixture (g/kg diet in cellulose): calcium carbonate (40% Ca) 0.5 g, magnesium oxide (60% Mg) 1.24 g, ferric citrate 0.2 g, potassium iodide (75% I) 4 mg, zinc sulfate (36% Zn) 0.4 g, copper sulfate (25% Cu) 0.3 g, manganese sulfate (33% Mn) 0.3 g, dibasic calcium phosphate (23% Ca, 18% P) 2 g, cobalt sulfate 2 mg, potassium hydrogen phosphate (28% K; 22% P) 3 g sodium selenite (30% Se) 3 mg, Sodium chloride 0.4 g. Vitamin mixture (mg/kg diet in cellulose): DL-α tocopherol acetate 100 mg, retinyl acetate 0.75 mg DL cholecalciferol 6 mg, thiamin 10 mg, riboflavin 15 mg, pyridoxine 10 mg, vit. B 0.05 mg, nicotinic acid 10 mg, folic acid 0.2 mg, inositol 500 mg, biotin 0.1 mg, calcium panthotenate 20 mg, Cyanocobalamine 0.02 mg, choline chloride 1000 mg.

### 2.2 Experimental design and feeding of fish

The study was carried out at the golden mahseer hatchery facility of ICAR-DCFR, Bhimtal, in a flow-through system. For this, 144 golden mahseer, *Tor putitora* (Hamilton, 1822) juveniles were assigned into four treatment groups at random in triplicates. Twelve mahseer juveniles (av. weight 6.33 ± 0.1 g) were placed in every replicate rectangular fiber-reinforced plastic (FRP) tray (46.0 cm x 46.0 cm x 19.5 cm; length, width, and depth) with a meshed-bottom sheet for water movement. Three such replicate trays were kept in an FRP trough (partially submerged; 220.0 cm x 50.0 cm x 40.0 cm) for each treatment group. Prior to the trial, fish were acclimated in the experimental unit for 30 days on the basal diet.

Fish in each treatment group were fed with their respective diet *ad libitum* for five weeks post acclimation. Any uneaten feed and fecal waste was siphoned every morning. A moderate water flow (7–10 L/hour) was maintained in each trough, and aerators were used for maintaining dissolved oxygen (DO). The quality of water was checked on a regular basis (pH: 7.3−7.8; temperature: 22.1–23.4 °C; DO: 5.8–6.4 mg/L; total hardness: 124–139 mg/L; alkalinity: 73–86 mg/L; ammonia: <0.01 mg/L and nitrite: <0.1 mg/L). A multi-parameter water testing system was used to keep track of temperature, DO, and pH (HQ40d, Hach, USA). While, commercially available kits were used to measure alkalinity, total hardness, ammonia, and nitrite (HiMedia, India). After five weeks of feeding, the fish were batch weighed and weight gain percentage was calculated using the formula

Weightgain(%)=[(Finalweight−Initialweight)/Initialweight]x100

### 2.3 Ammonia challenge

After the feeding trial, the water flow in experimental units/troughs was stopped, and golden mahseer juveniles in each treatment (16 juveniles each, in duplicates) were exposed to ammonia-nitrogen (total ammonium nitrogen (TAN) concentration of 10 mg L^−1^) for 96 h. The group that received the control diet (0% β-glucan) was split into two groups: one served as control, and the other was exposed to ammonia. A stock solution of NH_4_Cl (1000 mg/L) was used as a source of TAN, and an appropriate volume of the freshly made stock solution was added to get the desired concentration in the experimental units. Throughout the ammonia challenge trial, each tank's total water volume was kept at 300 L. Continuous aeration was ensured to provide the optimum DO, and feeding was stopped during the challenge period. Every morning, the fecal matter was removed by siphoning to reduce ammonia buildup, and around 75% water of the units was replenished, and TAN levels were adjusted by adding the required volume of NH_4_Cl stock solution. Further, the TAN levels were measured frequently [24] to avoid differences between nominal and actual TAN concentrations in the units. After 96 h of ammonia exposure, fish were sampled for gene expression study.

### 2.4 Sampling

After 96 h of ammonia exposure, six juveniles (*n* = 6 each) from different treatment groups were randomly collected, anaesthetized (50 µL clove oil/L of water), and euthanized. The fish were then dissected out aseptically, and tissues samples (liver and gill) were collected and quickly frozen in liquid nitrogen for further gene expression study.

### 2.5 Quantitative expression of various genes

TRIzol reagent (Invitrogen, USA) was used to extract total RNA (as per the manufacturer's recommendations). A spectrophotometer (Biotek, VT, USA) and agarose gel electrophoresis were used to evaluate the amount and quality of the extracted RNA, respectively. The PrimeScript First-Strand cDNA synthesis kit was then used to reverse transcribe the RNA and create complementary DNA (cDNA) (Takara, USA). Real-time qPCR was conducted using the synthesized cDNA as a template. The relative expression levels of targeted genes namely catalase (*cat*), superoxide dismutase (*sod*), glutathione-s-transferase (*gst*), inducible nitric oxide synthase (*inos*), toll-like receptor 4 (*tlr4*), toll-like receptor 5 (*tlr5*), interleukin-1β (*il-1β*), complement factor 3 (*c3*), immunoglobulin light chain (*igl*), major histocompatibility complex 1 (mch1), and aquaporins (*aqp* namely aquaporins 1a: *aqp1a*; aquaporins 3a: *aqp3a;* and aquaporins 3b: *aqp3b*) were evaluated in a qPCR machine (Applied Biosystem, USA) using SYBRPremix Ex TaqII (Takara, USA) and gene-specific primers (designed according to the method described by Thornton and Basu [25], using Primer 3.0 (http://primer3.ut.ee/) software, Table 2) under standardized reaction conditions [21]. Then, utilizing 18S ribosomal RNA (18 s) as the reference gene, the relative expression (fold-change) of the various genes was assessed using Pfaffl's [26] mathematical model.

**Table 2:** Real-time qPCR primers.

Primers	Sequence (5′−3′)	Ta (°C)	Amplification efficiency (%)	Amplicon size (bp)	Accession no.
*cat*	F- CCGATGAGGGCAACTGGGAT R-TGAGAGTGGATGAAGGACGGAA	60	106.9%	91	MG821473.1
*sod1*	F- GGCACCGTTCATTTCGAGCA R-TGATGCAGCCGTTTGTGTTGT	60	101.3%	130	KY569539.1
*gst*	F-AGAGGGAAGATGGAGTCGGT R-AACCAAAGGCACCTGCTGAAA	60	101.9%	138	XM_026224781.1
*inos*	F-AGGTGGCAGAGAGATGAACGAA R-AGGAGGCTTTGTGAGGGTGG	60	103.8%	95	HQ589354.1
*il1β*	F-CAACCTGTGTGCCTGGGAAT R-CTCGTTCGGGTCATCGGCTTT	60	107.5%	135	MN193586.1
*tlr4*	F-GCGGGACTTTCAAGCAGGGA R-AGCGACACCAGGCACTATCAA	60	107.6%	119	LC441112.1
*tlr5*	F- CTGATCCTCAGGACTGGCAC R- GTTCCGTTGTGACTGCAACC	60	104.6%	109	XP_016373368
*mhc1*	F- GTTTTGCCCTGGTGTTCCAC R- CTTCCTCGTCTCCAGTCACG	60	99.9%	220	AFO38429
*c3*	F-AGCAGGAGGTGGAAGGGACA R-CAGGTTTGGGACACTCAGGCA	60	106.9%	113	MN531579.2
*igl*	F- GTGTGTGGCCAGTAAGGGAT R- ACCGGGACTCAGATTGACAC	60	109.1%	94	BAB90985
*aqp1a*	F-GCGAGCGGTATCGTGTATGG R-AGCTGGAAGGTAGCGAAGAGT	60	97.1	116	LC069004.1
*aqp3a*	F-GGCAAAGGAAGACATCAGGACC R-CCACAACCAAACATCACCAGGA	60	97.8	154	LC069008.1
*aqp3b*	F-CCCAGTTGATTCTAAGCGGAGG R-GATTCCCAATGTAGCGGCGAA	60	101.2	83	LC069010.2
*18s*	F-ATTGACGGAAGGGCACCACC R-CAGACAAATCGCTCCACCAACT	60	102.47%	166	SRX2442156

### 2.6 Statistical analysis

The data were subjected to a one-way analysis of variance using the statistical program SPSS (Version 19.0), then Tukey's post-hoc multiple comparisons. A probability value of less than 0.05 was used to determine statistical significance. The data is presented as a mean with standard error (SE).

### 2.7 Ethical statement

The Institutional Animal Ethics Committee at the ICAR-DCFR, Bhimtal, Nainital, Uttarakhand, provided the guidelines for all of the experimental and sampling techniques used in this study.

## 3 Results

### 3.1 Growth of mahseer juveniles

**Fig. 1:**
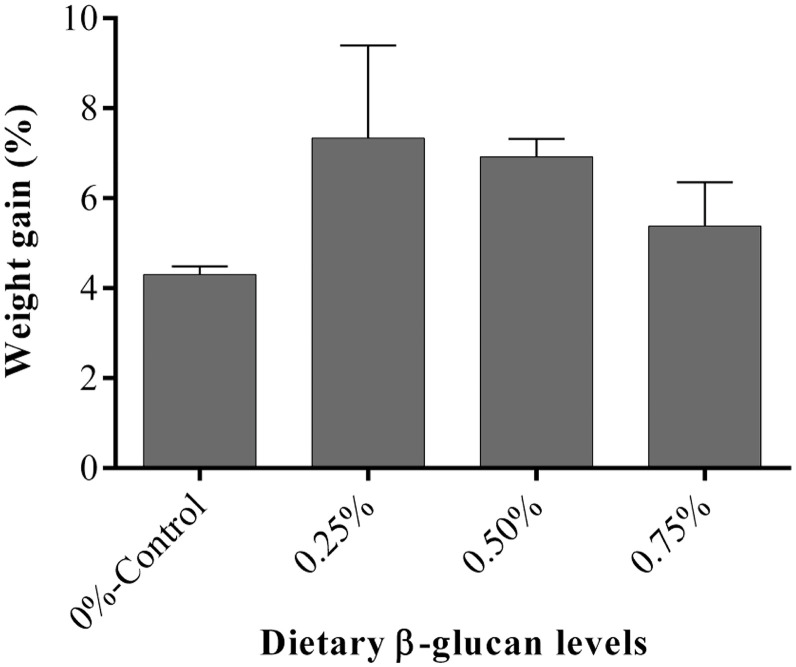
Effect of dietary β-glucan supplementation on growth performance of golden mahseer juveniles. Data are expressed as Mean ± SE, *n* = 36.

Dietary intake of β-glucan for five weeks had no significant influence on the weight gain (%) of golden mahseer compared to those fed on the basal diet (Fig. 1). Although there was no statistically significant difference, the fish fed β-glucan (0.25–0.75%) reported a somewhat greater mean weight gain percentage than the control group.

### 3.2 Differential expression of genes encoding anti-oxidative enzymes and inducible nitric oxide synthase (*inos*) of golden mahseer juveniles exposed to ammonia

**Fig. 2:**
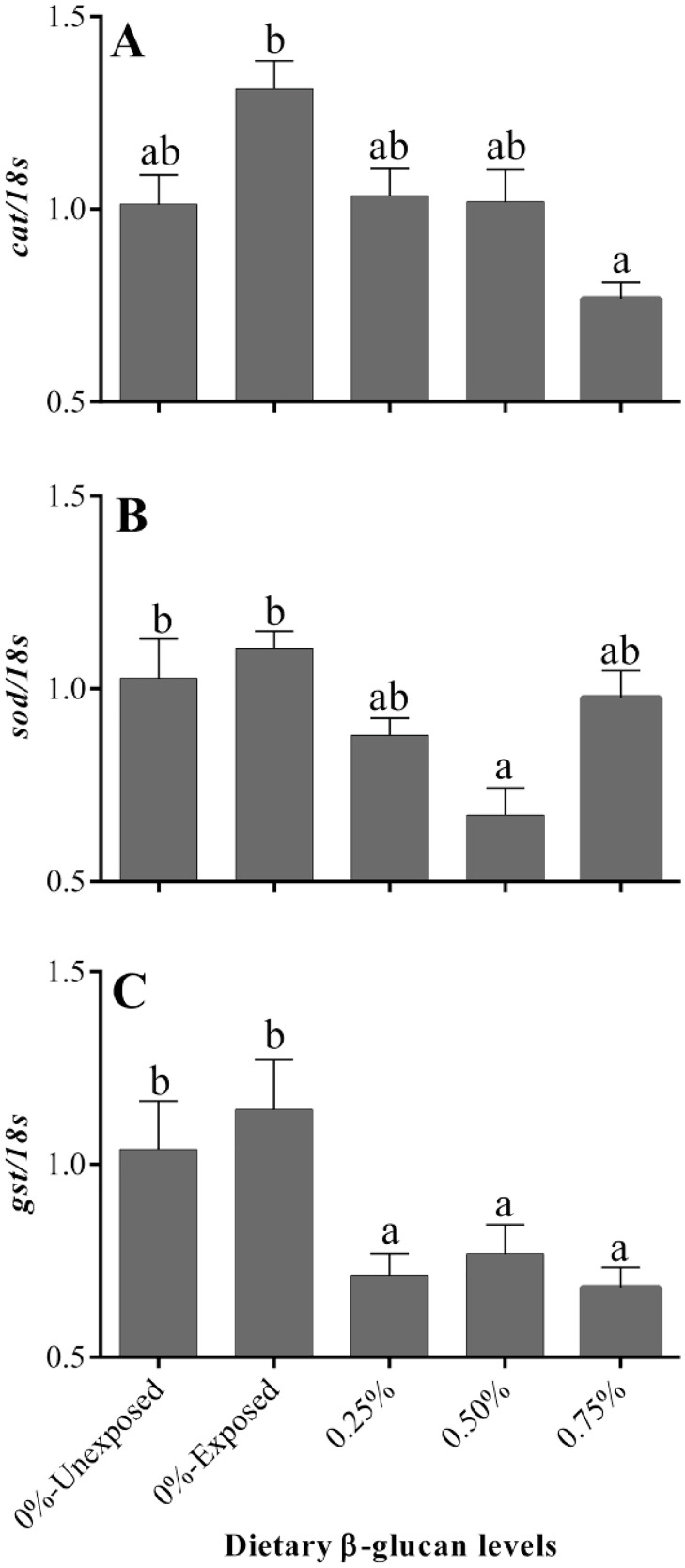
Effect of dietary β-glucan supplementation on the branchial expression of genes encoding anti-oxidative enzymes of golden mahseer juveniles exposed to ammonia. Different superscripts (a, b) above the bars in each panel, if any, indicate significant difference (*p* < 0.05). Data are expressed as Mean ± SE, *n* = 6.

**Fig. 3:**
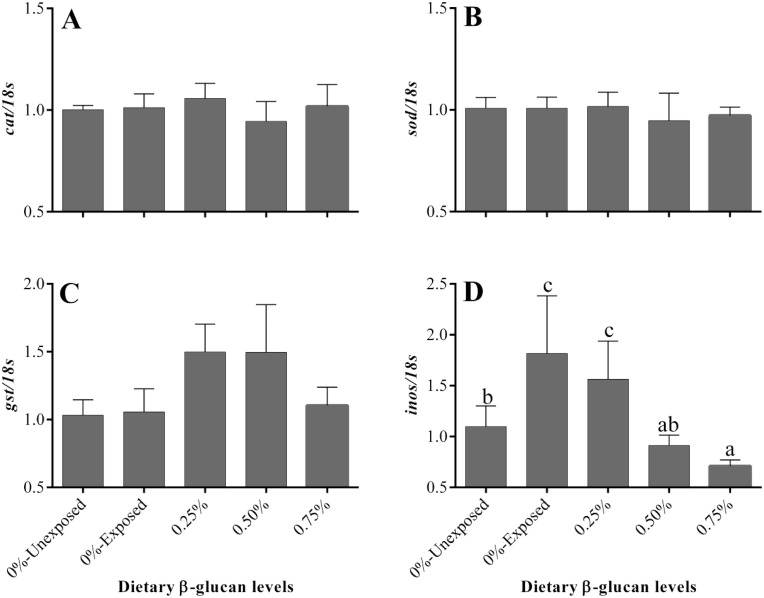
Effect of dietary β-glucan supplementation on the hepatic expression of genes encoding anti-oxidative enzymes and inducible nitric oxide synthase (*inos*) of golden mahseer juveniles exposed to ammonia. Different superscripts (a, b, c) above the bars in each panel, if any, indicate significant difference (*p* < 0.05). Data are expressed as Mean ± SE, *n* = 6.

Feeding β-glucan differentially modulated the mRNA levels of anti-oxidative genes in gill and liver tissues of ammonia-exposed golden mahseer juveniles. In gills, the abundance of antioxidative (catalase, superoxide dismutase, and glutathione-s-transferase) genes was significantly higher in ammonia-challenged fish that received a control diet, and β-glucan intake down-regulated their expression in different magnitudes (Fig. 2). For instance, the transcript abundance of branchial catalase was lowest in fish that received 0.75% β-glucan, while all the β-glucan fed fish irrespective of their intake levels recorded significantly the lowest mRNA levels of *gst* in the gill. On the other hand, the hepatic expression of all the studied antioxidative (*cat, sod*, and *gst*) genes were not found to vary among the various dietary groups exposed to ammonia (Fig. 3). Likewise, in the liver, transcript levels of *inos* were significantly higher in control and 0.25% β-glucan fed groups after 96 h of ammonia challenge. Higher intake of β-glucan (0.5 and 0.75%) reduced the abundance of hepatic *inos* (Fig. 3).

### 3.3 Immune genes expression in the liver of golden mahseer juveniles exposed to ammonia

**Fig. 4:**
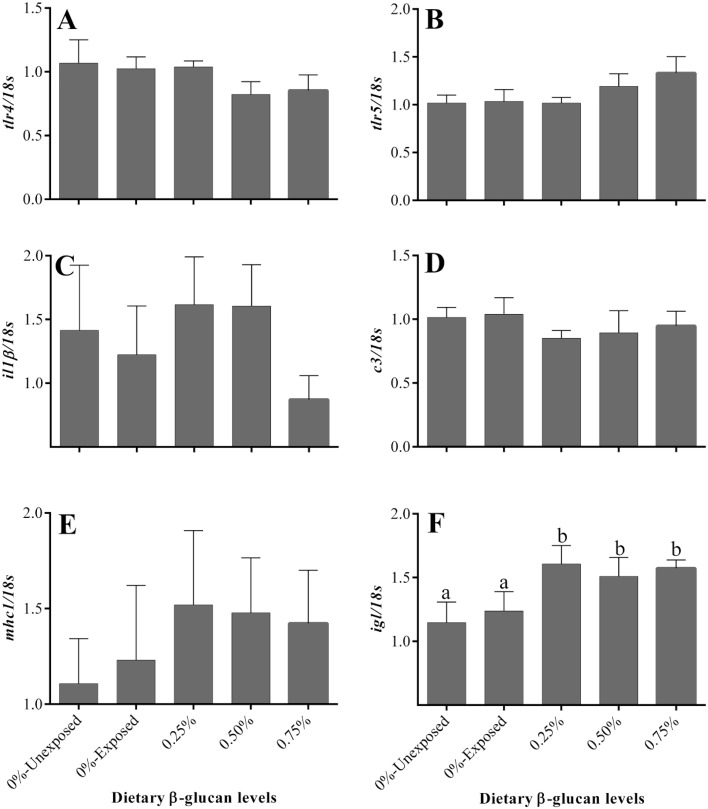
Effect of dietary β-glucan supplementation on the hepatic expression of immune genes of golden mahseer juveniles exposed to ammonia. Different superscripts (a, b) above the bars in each panel, if any, indicate significant difference (*p* < 0.05). Data are expressed as Mean ± SE, *n* = 6.

To study the effect of β-glucan intake on ammonia stress tolerance, we analyzed the mRNA expression levels of several immune genes in the liver. The transcript abundance of toll-like receptors (*tlr4* and *tlr5*), interleukin-1β (*il-1β*), complement factor 3 (*c3*), and major histocompatibility complex 1 (*mhc1*) were not significantly varied among the different dietary groups after 96 h of ammonia challenge (Fig. 4A-E). Conversely, the mRNA expression of *igl* was significantly upregulated in all the β-glucan fed groups (0.25, 0.5 and 0.75%) following the 96 h ammonia challenge (Fig. 4F). Although there was no statistically significant difference, the fish fed β-glucan (0.25–0.75%) recorded slightly greater transcript levels of *mhc1* than the control group with higher inter-individual variation.

### 3.4 Differential expression of aquaporins in ammonia-challenged golden mahseer juveniles

**Fig. 5:**
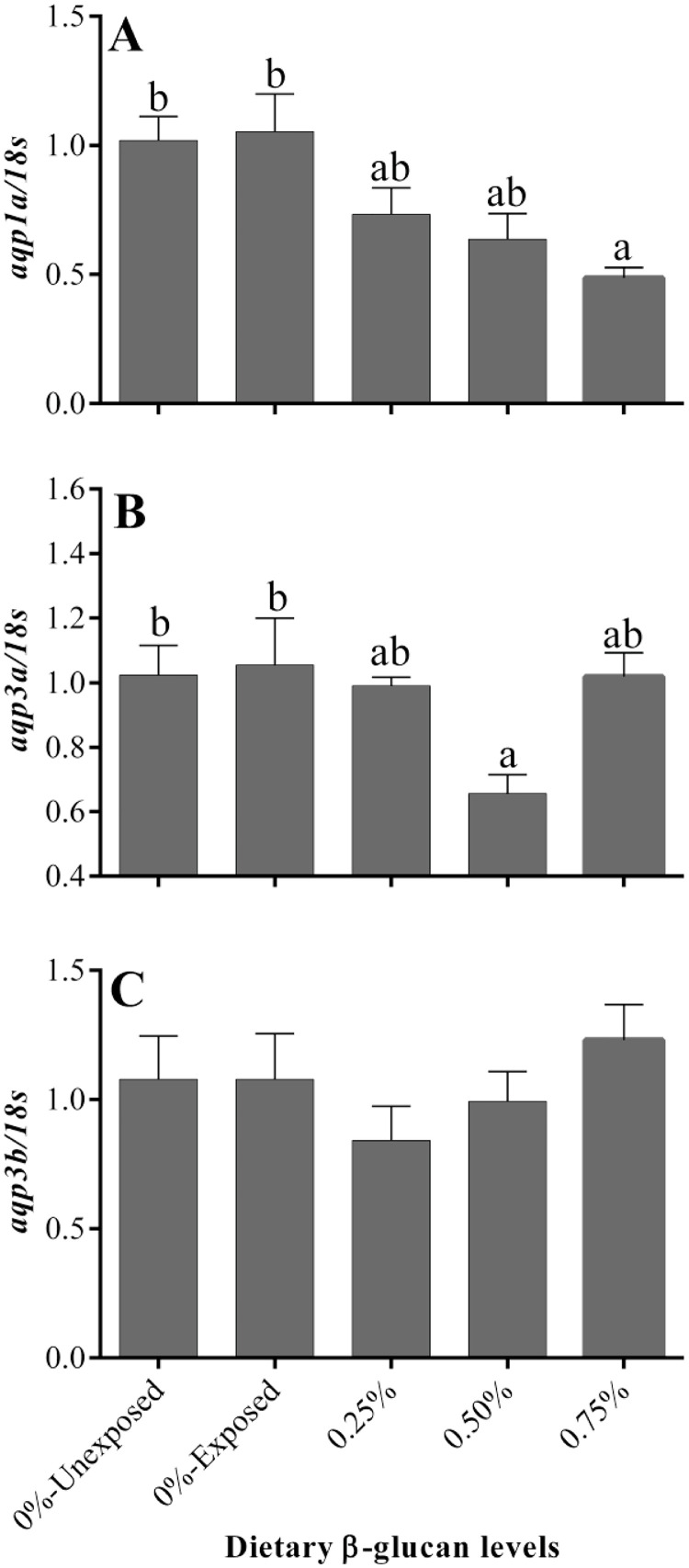
Effect of dietary β-glucan supplementation on the branchial expression of aquaporins of golden mahseer juveniles exposed to ammonia. Different superscripts (a, b) above the bars in each panel, if any, indicate significant difference (*p* < 0.05). Data are expressed as Mean ± SE, *n* = 6.

β-Glucan intake differentially modulated the transcript abundance of branchial aquaporins in ammonia-exposed golden mahseer juveniles. The highest expression of branchial aquaporin 1a and 3a was noticed in ammonia-challenged fish that received the control diet, and β-glucan supplementation significantly reduced their transcript abundance (Fig. 5A and B). Among the β-glucan dietary groups, the lowest mRNA abundance of aquaporin 1a was noticed in ammonia-challenged fish fed with 0.75% β-glucan. On the other hand, similar mRNA expression of aquaporin 3b was evidenced in the gill of ammonia-challenged groups irrespective of their β-glucan intake levels (Fig. 5C).

## 4 Discussion

It is known that functional feed additives and adequate nutrition stimulate fish growth and immune system and potentiate tolerance to different husbandry stressors in aquaculture. Although β-glucan has shown promise in increasing immunity and stress resistance, the results of different studies are highly variable and inconsistent depending upon the type of stressor, fish species, β-glucan source, feeding duration, dose etc. [27]. Nevertheless, little is known about its effect on the expression of water channel proteins/aquaporins and nitric oxide synthase during the ammonia challenge. Hence, in this study, we assessed the effects of dietary supplementation of β-glucan, sourced from *Saccharomyces cerevisiae,* on antioxidative, immune, and aquaporin gene expression in golden mahseer juveniles exposed to ammonia stress for 96 h.

In the current study, β-glucan administration had no significant effect on the weight gain (%) of golden mahseer juveniles. Our findings are in line with earlier studies on mahseer, grass carp, sea bass, and Nile tilapia, where glucan supplementation had little to no effect on growth performance [21,[28], [29], [30], [31]]. The administration of β-glucan, on the other hand, has been shown to enhance growth in a few other species [32], [33], [34], [35]. Previous studies have indicated that hyper-ammonia stress may generate free radicals such as ROS, RNS, and NO^.^, imparting oxidative stress [1,7]. The free radicals/ROS produced under stressful conditions can be eliminated by an enzyme system consisting of superoxide dismutase (SOD), catalase, glutathione-s-transferase, etc. [10]. Hence, natural anti-oxidants or compounds with anti-oxidant characteristics have been widely used to ameliorate the deleterious effects of stressors/ROS. In this context, the anti-oxidative capacity of β-glucan is well elucidated in various animal models suggests that glucan can serve as a powerful free-radical scavenger, and that macrophages specifically phagocytose and sequester glucan through its receptors on macrophages [36,37]. In this study, the free radical scavenging potential of β-glucan might have resulted in significant down-regulation of various anti-oxidative defense genes in the gills of ammonia-challenged fish, which is consistent with earlier reports of Kayali et al. [36]. Similarly, β-glucan shown to reduce hypoxia-induced oxidative damage in yellow croaker by reducing ROS production [38]. On the contrary, significantly higher activities of antioxidative enzymes were documented in β-glucan fed tilapia and Pacific white shrimp upon ammonia stress [39,40]. The increased sensitivity of the gill to water pollutants, such as ammonia, as it is the organ that is in direct contact with the aquatic environment and the stressor, is a plausible explanation for the significant changes in the transcript abundance of branchial anti-oxidant genes compared to the liver.

Concerning inducible nitric oxide synthase (*inos*), ammonia exposure upregulated its hepatic expression in fish that received a basal diet. Like our observation, induction of *inos,* leading to increased production of NO, has been reported in catfish and freshwater prawn under ammonia stress [8,9,41,42]. They believed that induction of *inos* leads to higher endogenous production of NO as a protective strategy to counteract ammonia-induced oxidative stress. But, NO at higher levels, induce nitrosative stress and favors cell cycle arrest and apoptosis [43]. In the present study, glucan intake (0.5 and 0.75%) was shown to reduce NO production in ammonia-challenged golden mahseer juveniles by down-regulating the transcript abundance of *inos* at par with the unexposed fish, indicating a low amount of oxidative/nitrosative stress in these glucan fed groups. A similar reduction in mRNA levels of *inos* was recently reported in the catfish Pangasianodon hypophthalmus exposed to multiple stressors and fed with diets supplemented with zinc nanoparticles [44]. However, in-depth studies are further necessary to understand the precise molecular mechanism by which glucan down-regulates *inos* expression.

Studies showed that immunological markers such as cytokines, c3 proteins, toll-like receptors, etc. are sensitive to stress [45,46]. Further, exposure to environmental toxicants/pollutants known to affect the expression of immune genes following oxidative stress and the release of pro-inflammatory mediators through the toll-like receptor signaling pathway [46]. In the present study, different groups fed with graded levels of β-glucan when exposed to ammonia showed no substantial difference in the expression levels of the immune genes studied, except for the *igl*. This may be explained by the fact that a short duration (96 h) of sub-lethal ammonia exposure may be insufficient to influence/trigger the immune system at the molecular level. Presumably, the increased mRNA levels of *igl* in β-glucan fed fish after ammonia challenge may be attributable to the increased antibody production in these groups. Our results are in line with an earlier study in carp wherein the authors reported a decline in immunoglobulin production and the number of IgM secreting cells in response to stress [47]. Further, a reduction in albumin:globulin ratio was reported in stressed *Labeo rohita* administered with β-glucan suggesting increased immunoglobulin production [48]. Earlier studies reported that the duration of exposure to stressors (acute and chronic stress) affects the fish immune system differently [49,50]. Similarly, innate immune markers like lysozyme and total serum protein concentrations were not significantly influenced by β-glucan in Nile tilapia exposed to hypoxia for nine hours [51]. On the other hand, the significant upregulation of *igl* in all the β-glucan fed ammonia-challenged groups suggests that ammonia-induced stress activates immunoglobulin synthesis in golden mahseer juveniles fed with β-glucan. It has already been documented that β-glucan can influence immunoglobulins synthesis in stressed/infected fish [52,53].

Aquaporins are aqueous channel proteins that play key roles in osmoregulation by selectively permeating solute molecules such as glycerol, urea, and ammonia [54,55]. According to studies, aquaporins are responsive to ammonia stress and hence ammonia tolerance in fish is linked to aquaporin 1 and 3 [56], [57], [58], [59]. In the present study, significantly low branchial mRNA expression of *aqp1*a and *aqp3a* was evidenced in glucan-fed fish when exposed to ammonia, suggesting a reduction of ammonia uptake from the water. Similar to our findings, Ip et al. [57] observed significant reductions in *aqp1aa* mRNA expression in the gills and skin of ammonia-exposed *Anabas testudineus* as a protective strategy to reduce ammonia influx. A similar reduction of *aqp3* mRNA levels was observed in several fish species when transferred from freshwater to seawater [60], [61], [62]. Conversely, few researchers reported upregulation of branchial *aqp3* in ammonia-exposed fish to facilitate ammonia excretion [58,63]. In the present study, the branchial *aqp3b* expression remained unchanged in different ammonia-exposed groups regardless of glucan intake. The mechanism by which β-glucan regulates the expression of aquaporins in golden mahseer exposed to environmental ammonia is still unclear, and further studies are necessary.

## 5 Conclusion

Our study demonstrated that β-glucan supplementation minimized the effect of ammonia-induced stress in golden mahseer juveniles as shown by the low transcription of genes involved in antioxidative defense and NO production. Further, glucan intake down-regulated branchial expression of *aqp1a* and *aqp3a* suggesting reduced ammonia uptake. On the other hand, mRNA expression of most of the studied immune genes was not significantly affected by β-glucan intake in ammonia-challenged fish. Taken together, this study showed that dietary intake of β-glucan improved resistance to ammonia stress to a certain degree, probably through activating the anti-oxidative system and reducing NO production via down regulating *inos*.

## Funding

The study was financially supported by the Department of Biotechnology (DBT), Ministry of Science and Technology, Government of India (vide reference no. BT/PR26920/AAQ/3/884/2017).

## CRediT authorship contribution statement

**Alexander Ciji:** Conceptualization, Investigation, Formal analysis, Writing – original draft. **Priyanka H. Tripathi:** Formal analysis. **Anupam Pandey:** Formal analysis. **Md Shahbaz Akhtar:** Conceptualization, Funding acquisition, Investigation, Data curation, Formal analysis, Writing – review & editing.

## Declaration of Competing Interest

The authors declare that they have no known competing financial interests or personal relationships that could have appeared to influence the work reported in this paper.

## Data Availability

Data will be made available on request.
